# Koumine exerts its anti-colorectal cancer effects by disrupting the interaction between HSP90 and CDC37, thereby downregulating downstream signaling pathways

**DOI:** 10.3389/fonc.2025.1687690

**Published:** 2026-01-19

**Authors:** HaiLing Lin, YuXuan Bao, XiTong Cheng, ShuMing Zhang, DanYang Zhou, WanCai Que

**Affiliations:** 1 Department of Pharmacy, Fujian Medical University Union Hospital, Fuzhou, China; 2 Phase I Clinical Trial Unit, Fujian Medical University Union Hospital, Fuzhou, China; 3 Department of Respiratory, Nanjing First Hospital, Nanjing Medical University, Nanjing, Jiangsu, China

**Keywords:** CDC37, colorectal cancer, HSP90, koumine, network pharmacology

## Abstract

**Background:**

Koumine, a principal bioactive alkaloid derived from the traditional Chinese herb *Gelsemium elegans*, has demonstrated broad cytotoxic activity against various cancer cell lines. However, its specific anti-tumor efficacy and underlying molecular mechanisms in colorectal cancer (CRC) remain largely unexplored.

**Methods:**

We employed an integrated strategy combining network pharmacology prediction with experimental validation. Bioinformatics analysis was conducted to identify potential targets. In vitro functional assays were performed to evaluate effects on cell proliferation, clonogenicity, apoptosis, migration, and invasion. Target engagement was confirmed by cellular thermal shift assay (CETSA), and the molecular mechanism was investigated through Western blot and co-immunoprecipitation analyses.

**Results:**

Network pharmacology identified heat shock protein 90 (HSP90) as a key potential target, a finding supported by molecular docking simulations. Koumine significantly inhibited the malignant phenotypes of CRC cells. CETSA confirmed direct binding of koumine to HSP90. Mechanistically, koumine disrupted the functional interaction between HSP90 and its co-chaperone CDC37, leading to the downregulation and inactivation of critical downstream client proteins, including cyclin-dependent kinases CDK4 and CDK6.

**Discussion:**

These findings elucidate that koumine exerts potent anti-CRC effects primarily by targeting the HSP90–CDC37 chaperone complex and inhibiting the CDK4/6–Rb signaling axis. This study provides a robust mechanistic foundation and compelling preclinical evidence for the further development of koumine as a promising therapeutic agent for colorectal cancer.

## Introduction

Colorectal cancer (CRC) is the most common malignancy of the digestive tract and ranks among the top five cancers worldwide in terms of incidence and mortality (1). In 2020, there were 1,148,515 new cases and 576,858 deaths globally (1), posing a significant burden on public health systems. Current treatment modalities for colorectal cancer include surgery, chemotherapy, radiation therapy, endoscopic therapy, targeted therapy, and immunotherapy (2). However, due to the rapid progression of colorectal cancer, many patients are diagnosed at advanced stages or with metastatic disease, making curative treatment extremely challenging (3). Despite undergoing surgery, a significant proportion of patients experience disease recurrence or progression, highlighting the limitations of current strategies (4). These limitations underscore the critical need to explore novel therapeutic agents. In this context, natural products have emerged as a promising source of anticancer leads, as exemplified by yuanhuacine from Genkwa Flos, which exhibits potent anti-CRC activity by targeting cell cycle regulators such as PLK1 (5). Inspired by such discoveries, we turned our attention to koumine—a major alkaloid from *Gelsemium elegans*—to systematically evaluate its anti-CRC efficacy and elucidate its underlying molecular mechanisms, with the aim of identifying a potential candidate for future adjuvant therapy development.

*Gelsemium elegans*, commonly known as *Gelsemium*, is a highly toxic plant belonging to the Loganiaceae family. It possesses various pharmacological activities, including anti-inflammatory, analgesic, sedative, anxiolytic, dermatological, and anticancer effects (6, 7). However, its therapeutic dose is very close to its toxic dose, making accidental ingestion potentially fatal (8, 9). The primary active components of *Gelsemium* are alkaloids, with more than 120 identified and categorized into nine main types: koumine-type, gelsemine-type, methyl koumine-type, humantenine-type, sempervirine-type, sarpagine-type, geleboline-type, gelsemamide-type, and geleselegine-type. Most of these compounds are indole and hydroxyindole alkaloids (7, 10). The toxicity of these alkaloids varies considerably, with koumine-type and gelsemine-type alkaloids exhibiting relatively lower toxicity in *in vivo* studies (11).

Koumine, a major component of Gelsemium alkaloids, has garnered significant attention for its antitumor properties. Its cytotoxic activity has been validated *in vitro* against various human cancer cell lines, including myeloid leukemia HL-60, hepatocellular carcinoma SMMC-7721, lung cancer A-549, breast cancer MCF-7, colon cancer SW480, and human bronchial epithelial BEAS-2B cells (12, 13). Additionally, koumine has been reported to inhibit the proliferation of hepatocellular carcinoma cells and induce apoptosis (14). The NF-κB and ERK/p38 MAPK signaling pathways have been implicated in koumine’s biological effects, operating in a reactive oxygen species–dependent manner (14). However, the potential anticancer effects of koumine on colorectal cancer cell lines other than SW480, as well as the specific molecular mechanisms involved, remain to be further elucidated.

In this study, network pharmacology and molecular docking were applied to identify the potential targets of koumine in colorectal cancer. We then verified its antitumor effects through both *in vitro* cellular experiments and *in vivo* animal studies and preliminarily explored the underlying molecular mechanisms using cellular thermal shift assay (CETSA) and Western blot analyses. These results provide supportive evidence for koumine’s potential as a therapeutic candidate for colorectal cancer.

## Methods

### Targets screening of koumine and colorectal cancer

Potential gene targets associated with koumine were identified and aggregated using the SwissTargetPrediction tool (15), with Homo sapiens selected as the species of interest. Concurrently, human genes implicated in the pathology of colorectal cancer were systematically retrieved from the DisGeNET database (16). These target genes were subsequently mapped to UniProtKB IDs to remove redundancy (17). A Venn diagram was generated using the Venn plug-in to identify overlapping genes between koumine-related targets and colorectal cancer–associated genes.

### Pathway network construction

To elucidate the underlying mechanisms, gene co-expression patterns, and protein–protein interactions (PPIs), the overlapping genes were integrated into the STRING database (18), with the confidence score threshold set to 0.7 to ensure reliable PPI identification. The resulting network was imported into Cytoscape (19) software (version 3.9.1) for visualization. Network topological analysis was performed using the Network Analyzer tool, and the CytoHubba plug-in was applied to identify and characterize key hub genes within the network.

### Enrichment of GO function and KEGG pathway

Intersecting targets were imported into the Metascape database (http://www.metascape.org), with Homo sapiens selected as the species. The enrichment results were filtered using a P value threshold of ≤ 0.01. Gene ontology (GO) functional enrichment analysis was performed, including biological processes, cellular components, and molecular functions, along with Kyoto Encyclopedia of Genes and Genomes (KEGG) pathway enrichment analysis. For visualization, the top 20 significantly enriched terms from both GO and KEGG analyses were selected and ranked according to their P values.

### Molecular docking evaluation

The three-dimensional structures of receptor proteins and ligand molecules were obtained from the Protein Data Bank (PDB) and PubChem databases, respectively. Ligand molecules were converted to PDBQT format using OpenBabel version 3.1.1. Receptor structures were preprocessed using AutoDockTools version 1.5.6 to remove water molecules and add hydrogen atoms. Docking grid parameters were defined using the Grid module, with the docking mode set to semi-flexible docking and the docking algorithm specified as the Lamarckian genetic algorithm (**Supplementary Table S1**). Molecular docking was conducted using AutoDock Vina version 1.1.2 (20), and docking results were visualized using PyMOL version 2.20.

### Cell culture and treatment

Human colorectal cancer cell lines HCT15 and HCT116 were obtained from the Cell Bank of the Chinese Academy of Sciences. Cells were cultured in high-glucose DMEM supplemented with 10% fetal bovine serum (FBS; Gibco, USA) and 1% triple antibiotics and maintained at 37°C in a humidified incubator with 5% CO_2_. When cell confluency reached approximately 80%–90%, cells were digested and passaged. Koumine was purchased from Shanghai Yuanye Bio-Technology Co., Ltd., and prepared as a 10 mM stock solution in dimethyl sulfoxide (DMSO). For cell-based experiments, koumine was added to the culture medium at final concentrations of 100, 200, and 400 μg/mL and incubated for the indicated durations before subsequent assays. Oxaliplatin (L-OHP; S24033, Yuanye Bio) was used as a positive control at a concentration of 5 μg/mL.

### CCK8 staining

HCT15 and HCT116 cells in the logarithmic growth phase were seeded into 96-well plates at a density of 5 × 10³ cells per well. After treatment with koumine or L-OHP at the indicated concentrations, cells were incubated for 48 h. Subsequently, 10 μL of CCK-8 reagent (C0038, Beyotime, China) was added to each well, mixed gently, and incubated at 37°C with 5% CO_2_ for 1 h. Absorbance at 450 nm was measured using a microplate reader. Each experiment was performed in triplicate, and mean values were calculated.

### Colony assay

HCT15 and HCT116 cells in the logarithmic growth phase were seeded into 6-well plates at a density of 500 cells per well and incubated at 37°C with 5% CO_2_ for 14 days. During incubation, cells were treated with koumine or L-OHP as indicated. After 14 days, visible colonies were observed. The culture medium was removed, and wells were washed twice with precooled phosphate-buffered saline (PBS). Cells were fixed with 4% paraformaldehyde for 20 min, followed by staining with 1 mL of 5% crystal violet for 20 min. Wells were then washed twice with precooled PBS and air-dried. Colony numbers were subsequently counted.

### Cell apoptosis detection

After treatment of colorectal cancer cells with koumine or L-OHP for 48 h, cells were digested with trypsin without EDTA for 9 min and collected into centrifuge tubes. The cells were centrifuged at 500 × g for 5 min, the supernatant was discarded, and the cells were washed twice with precooled PBS. Each sample was resuspended in 400 μL of 1× Annexin V binding buffer, followed by the addition of 5 μL of Annexin V–FITC staining solution (CA1030, Solarbio, China) with gentle mixing. Samples were incubated on ice for 15 min, after which 5 μL of propidium iodide (PI) staining solution was added and incubated for an additional 5 min on ice. All procedures were performed in the dark. Apoptosis rates were subsequently analyzed by flow cytometry.

### Wound scratch assay

Cells in the logarithmic growth phase were digested, centrifuged, and resuspended to prepare a single-cell suspension. Cells were seeded into 6-well plates at a density of 5 × 10^5^ cells per well and cultured until confluency exceeded 90%. A straight scratch was created using a sterile 10 μL pipette tip. Wells were washed twice with PBS to remove detached cells, followed by the addition of serum-free medium. Images of the wound area were captured at 0 h and 24 h at the same location under a microscope. The wound healing rate was calculated as follows:


Cell scratch healing rate = [(scratch area at 0h − scratch area at 24h)/scratch area at 0 h] × 100%.

### Transwell assay

Matrigel (50 μL) was added to the upper chamber of each Transwell insert and allowed to solidify in a cell incubator at 37°C with 5% CO_2_ for 1.5 h. Logarithmically growing cells were adjusted to a density of 7 × 10³ cells per well. Subsequently, 100 μL of cell suspension containing koumine or L-OHP was added to the upper chamber. The lower chamber was filled with 600 μL of culture medium supplemented with 10% FBS. Plates were incubated at 37°C with 5% CO_2_ for 48 h. After incubation, media from both chambers were removed, and the upper chamber was gently washed twice with precooled PBS. Cells were fixed with 4% paraformaldehyde for 20 min and stained with 600 μL of 5% crystal violet for 20 min. Chambers were washed twice with precooled PBS, air-dried in an inverted position, and photographed under an inverted microscope. Migrated cells were counted by randomly selecting five fields of view at the same magnification, and mean values were calculated.

### Western blot assay

After treatment with koumine at the indicated concentrations or an equivalent volume of DMSO for 48 h, cells were scraped and collected into centrifuge tubes. Samples were centrifuged at 2500 rpm for 10 min, and the supernatant was discarded. Cell pellets were resuspended in RIPA lysis buffer and lysed on ice for 30 min, followed by centrifugation at 12,000 rpm for 30 min. The supernatants containing total protein were collected. Protein concentrations were determined using the bicinchoninic acid (BCA) assay, and equal amounts of protein were prepared with loading buffer. Proteins were separated on 7.5%, 10%, or 12% SDS-PAGE gels and transferred onto polyvinylidene difluoride (PVDF) membranes. Membranes were washed with TBST, blocked with 5% non-fat milk for 2 h, and incubated overnight at 4°C with primary antibodies (**Supplementary Table S2**). After incubation with secondary antibodies at room temperature for 2 h, membranes were washed and developed using enhanced chemiluminescence (ECL) reagents. Band intensities were quantified using ImageJ software.

### Cellular thermal shift assay

A 50 μL human washed platelet suspension was prepared in a 1.5 mL microcentrifuge tube and incubated with koumine at 37°C for 20 min. A thermostatic metal bath was preheated to the indicated temperatures. Both koumine-treated and untreated platelet suspensions were heated for 4 min, followed by the addition of an equal volume of platelet lysis buffer (50 mM Tris, pH 7.4, 150 mM NaCl, 1% Triton X-100, 1% sodium deoxycholate, and 0.1% SDS). Samples were vortexed, placed on ice, and centrifuged at 13,000 × g for 3 min at 4°C. The supernatants were transferred to new tubes and either stored at −80°C or prepared for protein analysis by Western blotting. Prior to electrophoresis, samples were mixed with 5× loading buffer at a 1:5 ratio and boiled for 10 min.

### Co-immunoprecipitation assay

To validate the predicted disruption of the HSP90–CDC37 interaction by koumine, co-immunoprecipitation assays were performed. HEK293T cells were transiently co-transfected with plasmids encoding Flag-tagged HSP90 and HA-tagged CDC37 using Lipofectamine 3000 according to the manufacturer’s instructions. After 24 h, cells were treated with 100 μg/mL koumine or vehicle control (0.1% DMSO) for an additional 12 h. Cells were harvested and lysed in IP lysis buffer (50 mM Tris-HCl, pH 7.4, 150 mM NaCl, 1% NP-40, 0.5% sodium deoxycholate) supplemented with protease and phosphatase inhibitors. Lysates were centrifuged at 12,000 × g for 15 min at 4°C, and protein concentrations were determined using the BCA assay. For each immunoprecipitation, 500 μg of total protein was incubated with anti-Flag magnetic beads (P2115, Beyotime, China) overnight at 4°C with gentle rotation. Beads were washed five times with cold IP lysis buffer, and bound proteins were eluted by boiling in 2× Laemmli buffer for 10 min at 95°C. Input and immunoprecipitated samples were analyzed by SDS-PAGE followed by Western blotting.

### *In vivo* xenograft tumor model

Eighteen female BALB/c nude mice (4–6 weeks old) were purchased from Shanghai Ji Hui Laboratory Animal Co., Ltd. All animal experiments were approved by the Animal Care and Use Committee of Fujian Medical University (Approval No.: IACUC FJMU 2025-0292) and conducted in accordance with institutional guidelines. Mice were housed under specific pathogen-free conditions with controlled temperature and humidity and provided ad libitum access to food and water. HCT116 cells were harvested and resuspended in a 1:1 mixture of serum-free medium and Matrigel at a density of 5 × 10^6^ cells per 200 μL. Cell suspensions were subcutaneously injected into the dorsal region of each mouse. When average tumor volumes reached approximately 100 mm³ (10 days post-inoculation), mice were randomly assigned to three groups (n = 6 per group): (i) model group (vehicle control), (ii) L-OHP group (8 mg/kg, intraperitoneal injection every 2 days), and (iii) KM group (10 mg/kg, intraperitoneal injection daily). Treatments were administered for 28 days. Tumor size was measured every 3 days using calipers, and tumor volume was calculated as follows: V = Length × Width² × 0.52. Body weight was monitored throughout the study. At the end of the experiment, mice were euthanized in accordance with ethical guidelines, and tumors were excised, weighed, photographed, fixed in 4% paraformaldehyde for histological analysis.

### Histopathological and immunohistochemical analysis

Excised tumor tissues were fixed in 4% paraformaldehyde, dehydrated, and embedded in paraffin. Sections with a thickness of 4 μm were cut and mounted on slides. For hematoxylin and eosin (H&E) staining, sections were deparaffinized, rehydrated, stained with hematoxylin and eosin (G1005, ServiceBio, China), and examined under a light microscope to assess tissue morphology and pathological changes. For immunohistochemical (IHC) analysis, antigen retrieval was performed using citrate buffer, and endogenous peroxidase activity was blocked with 3% hydrogen peroxide. Sections were incubated overnight at 4°C with primary antibodies against caspase-3 and HSP90. After washing, sections were incubated with horseradish peroxidase (HRP)–conjugated secondary antibodies, developed using 3,3′-diaminobenzidine (DAB) substrate, and counterstained with hematoxylin. Stained sections were visualized and imaged using a light microscope.

### Statistics analysis

Cell-based experiments were performed at least three times. Data are presented as the mean ± SD. Statistical analyses were conducted using GraphPad Prism version 8.0. Comparisons between two groups were performed using Student’s t-test.

## Results

### Potential therapeutic targets of koumine in colorectal cancer treatment

**Figure 1:**
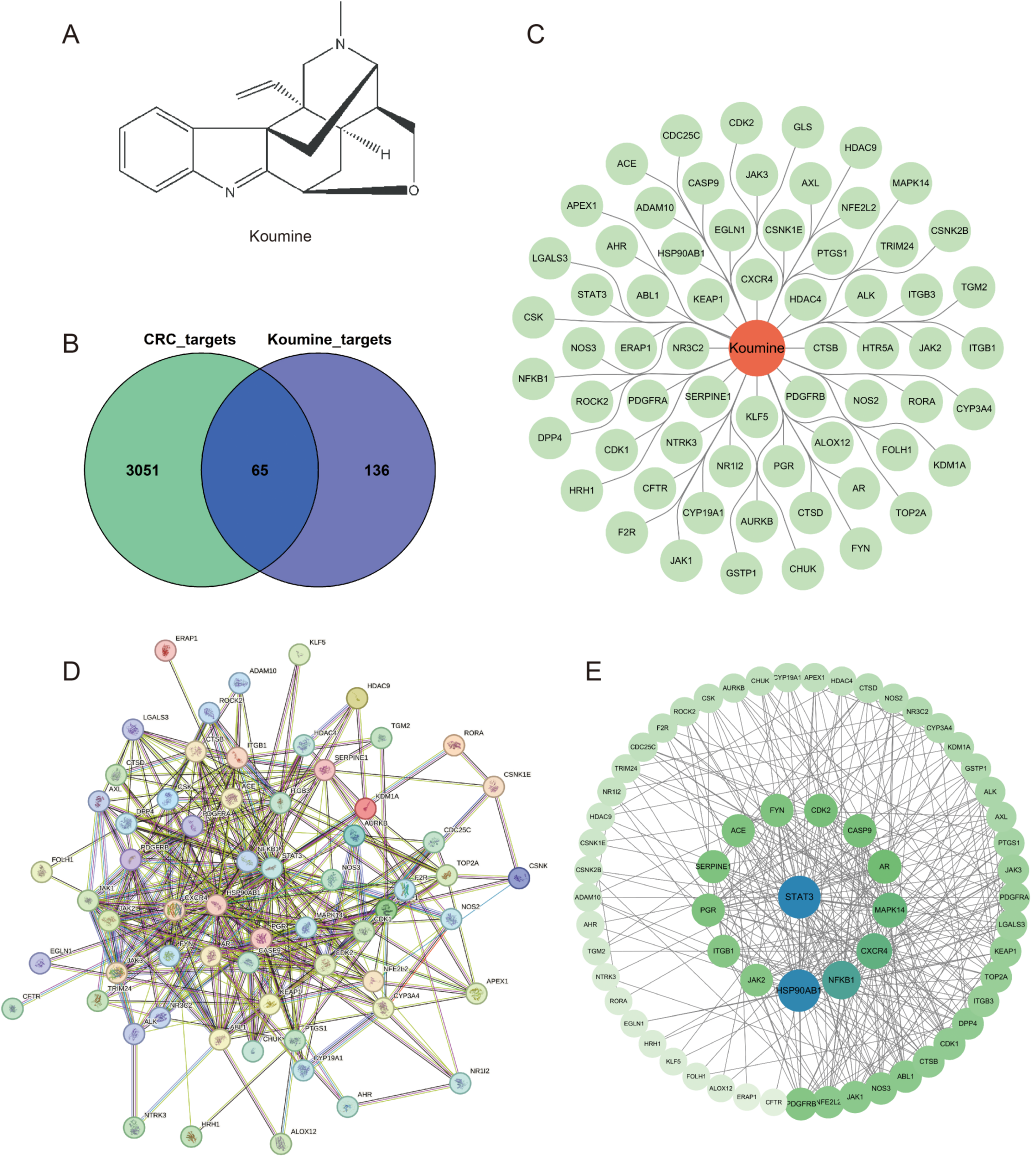
Identification and visualization of potential therapeutic targets of koumine for colorectal cancer. **(A)** Molecular structure of koumine. **(B)** Venn diagram showing the overlap between koumine-related targets and colorectal cancer–related targets. **(C)** Visualization of the 65 intersecting targets. **(D)** Protein–protein interaction (PPI) network of the potential therapeutic targets. **(E)** Key subnetwork of core targets identified through module analysis.

The chemical structure of koumine was obtained from the PubChem database and is shown in **Figure 1A**. A total of 201 potential targets of koumine were identified using the SwissTargetPrediction database, while 3117 colorectal cancer–related targets were retrieved from the DisGeNET database. Among these, 65 overlapping targets were identified between the drug and disease datasets, as illustrated in **Figure 1B**. The list of overlapping targets is shown in **Figure 1C**. These targets were subsequently imported into the STRING database for protein–protein interaction network analysis. After removing isolated nodes, the interaction network was visualized using Cytoscape (**Figure 1D**). Ranking of node degree values (**Figure 1E**) indicated that STAT3 (degree = 30,899) and HSP90AB1 (degree = 29,995) exhibited the highest interaction frequencies. Other targets, including NFKB1, CXCR4, MAPK14, AR, CASP9, CDK9, CDK2, FYN, ACE, SERPINE1, PGR, ITGB1, and JAK2, constituted a secondary interaction tier, suggesting a strong association between koumine and these signaling molecules.

**Figure 2:**
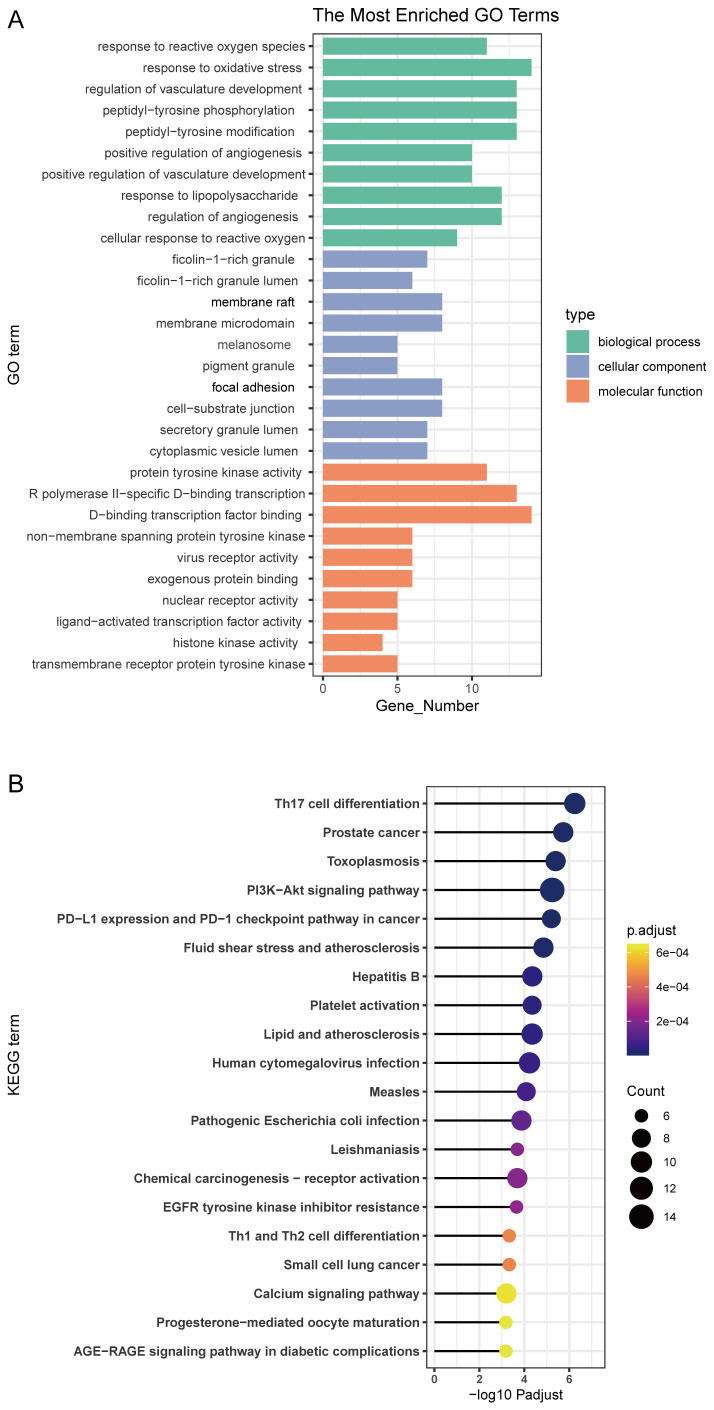
Enrichment analysis of target proteins. **(A)** Gene ontology (GO) enrichment analysis of the 65 overlapping targets between koumine and colorectal cancer, categorized into biological processes (green), cellular components (blue), and molecular functions (orange). Representative enriched GO terms include response to reactive oxygen species, regulation of angiogenesis, and transcription factor binding. **(B)** Kyoto Encyclopedia of Genes and Genomes (KEGG) pathway enrichment analysis of the same targets. Notably enriched pathways include the PI3K–Akt signaling pathway, PD-L1 expression and PD-1 checkpoint pathway in cancer, EGFR tyrosine kinase inhibitor resistance, and the calcium signaling pathway. Dot size represents the number of enriched genes, and color indicates the adjusted p-value.

Gene ontology (GO) functional annotation analysis was performed to further characterize the potential therapeutic targets of koumine in colorectal cancer. The top 10 enriched GO terms for biological processes (BP), cellular components (CC), and molecular functions (MF) are shown in **Figure 2A**. In the BP category, enrichment was mainly associated with response to reactive oxygen species, response to oxidative stress, and regulation of vasculature development. The CC category was primarily enriched in ficolin-1–rich granules, ficolin-1–rich granule lumen, and membrane rafts. For MF, significantly enriched terms included protein tyrosine kinase activity, RNA polymerase II–specific DNA-binding transcription factor activity, and DNA-binding transcription factor activity.

The top 20 enriched Kyoto Encyclopedia of Genes and Genomes (KEGG) pathways for the overlapping targets are presented in **Figure 2B**. Notably enriched pathways included the PI3K–Akt signaling pathway, PD-L1 expression and PD-1 checkpoint pathway in cancer, chemical carcinogenesis–receptor activation, EGFR tyrosine kinase inhibitor resistance, and the calcium signaling pathway, all of which play critical roles in tumor progression and antitumor therapy.

**Figure 3:**
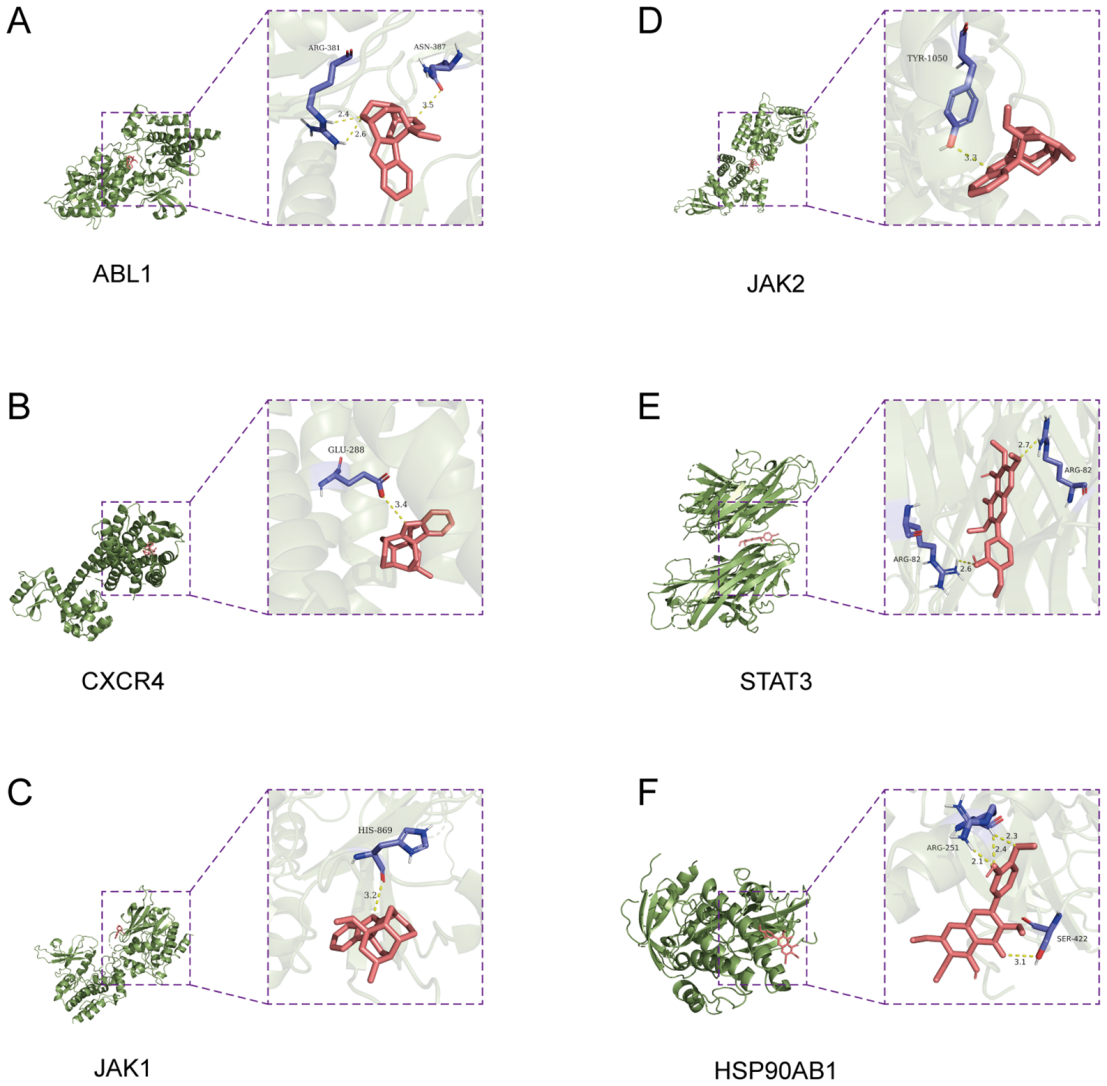
Molecular docking predicts interactions between koumine and potential targets. Predicted three-dimensional binding interactions between koumine (pink) and six potential target proteins: **(A)** ABL1, **(B)** CXCR4, **(C)** JAK1, **(D)** JAK2, **(E)** STAT3, and **(F)** HSP90AB1. Insets show detailed views of hydrogen bonding and binding site interactions (blue residues) within the active pockets of each protein. Docking results indicate that koumine fits well into the binding sites and forms stable interactions, supporting a potential multitarget mechanism against colorectal cancer.

Molecular docking analysis was performed to explore the potential binding interactions between koumine and key target proteins (**Table 1**). Hydrogen bond interactions between koumine and amino acid residues of the target proteins are illustrated in **Figure 3**. The binding free energies of koumine with ABL1, CXCR4, JAK2, JAK1, STAT3, and HSP90AB1 were −7.8, −7.3, −7.2, −6.8, −7.7, and −5.7 kcal/mol, respectively. Koumine exhibited favorable binding conformations within the active sites of these proteins, supporting the predicted interactions between koumine and its potential therapeutic targets and suggesting its efficacy in colorectal cancer treatment.

**Table 1:** The results of Molecular docking.

Target Name	PDB: ID	Ligand	Bond length	Affinity (KJ/mol)	Ki (µM)	pKi
ABL1	2g2h	ARG-381 ASN-387	2.4,2.6 3.5	-7.83±0.31	1.71(1.03–2.85)	5.77(5.55–5.99)
CXCR4	3oe0	GLU-288	3.4	-7.31±0.18	5.03(3.97–6.37)	5.30(5.20–5.40)
JAK2	2b7a	TYR-1050	3.3	-7.22±0.22	6.62(5.07–8.65)	5.18(5.06–5.30)
JAK1	6sm8	HIS-869	3.2	-6.86±0.23	14.0(10.8–18.1)	4.85(4.74–4.97)
STAT3	4ybm	ARG-82	2.6,2.7	-6.73±0.35	18.5(12.3–27.9)	4.73(4.55–4.91)
HSP90AB1	5fwk	ARG-251 SER-422	2.1,2.4,2.3 3.1	-5.79±0.19	93.5(75.5–115.8)	4.03(3.94–4.12)

### Koumine suppresses cell proliferation and induces apoptosis in colorectal cancer

**Figure 4:**
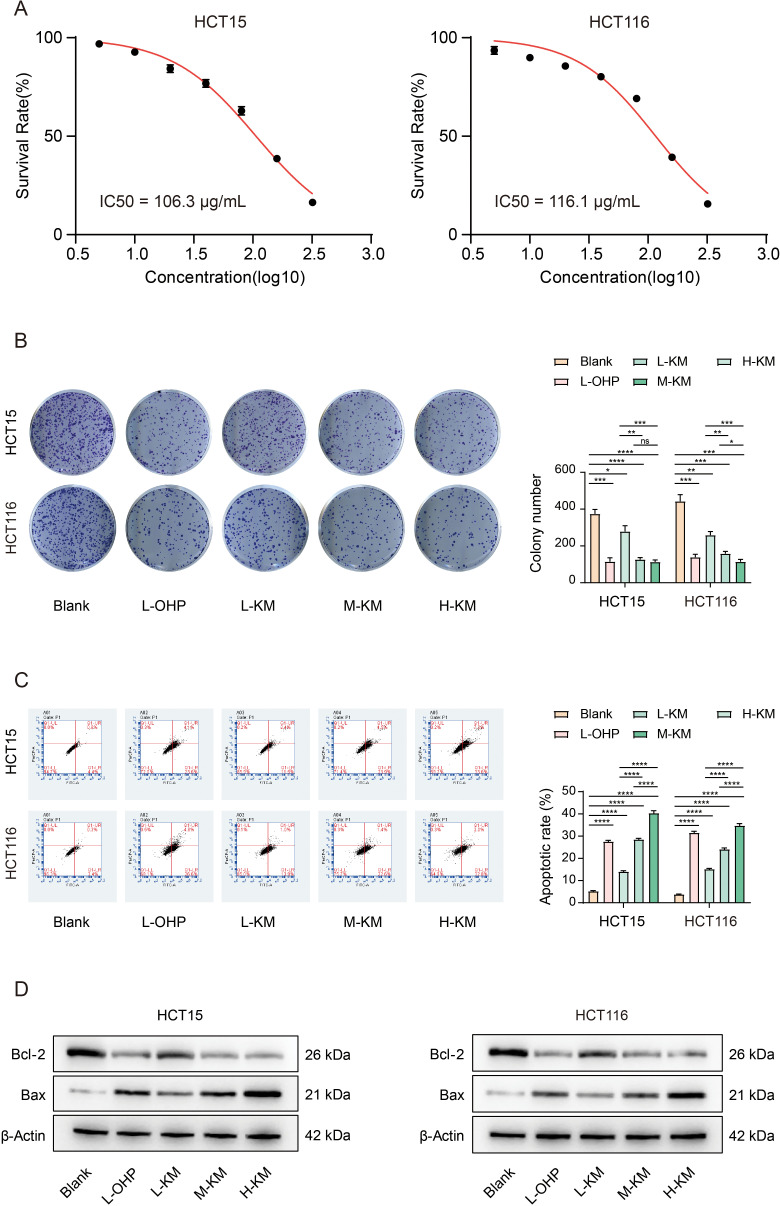
Koumine impairs cell proliferation and induces apoptosis in colorectal cancer cells. **(A)** Cell viability assessed by Cell Counting Kit-8 (CCK-8) assay following treatment with increasing concentrations of koumine. **(B)** Colony formation assay showing that koumine (100, 200, and 400 μg/mL) reduced colony formation in HCT15 and HCT116 cells in a dose-dependent manner compared with the control group. Oxaliplatin (L-OHP) was used as a positive control. Quantitative analysis is shown on the right. **(C)** Flow cytometric analysis of apoptosis in HCT15 and HCT116 cells treated with koumine (100, 200, and 400 μg/mL). **(D)** Western blot analysis of apoptosis-related proteins (Bcl-2 and Bax), showing increased Bax expression and decreased Bcl-2 expression following koumine treatment in both cell lines. β-Actin was used as a loading control. All experiments were independently repeated three times, and data are presented as mean ± SD. P < 0.05, *P < 0.01, **P < 0.001.

To further evaluate the anticancer effects of koumine on colorectal cancer cells, HCT15 and HCT116 cells were treated with koumine at final concentrations of 0, 5, 10, 20, 40, 80, 160, and 320 μg/mL and incubated for 48 h. Cell Counting Kit-8 (CCK-8) assays demonstrated that koumine significantly inhibited cell proliferation in a dose-dependent manner (**Figure 4A**). Curve fitting analysis revealed that the IC_50_ values of koumine were approximately 106.3 μg/mL for HCT15 cells and 116.1 μg/mL for HCT116 cells. To assess the effect of koumine on long-term proliferative capacity, colony formation assays were performed. As shown in **Figure 4B**, compared with the control group, the numbers of colonies formed by HCT15 and HCT116 cells were significantly reduced following treatment with low (Low-Koumine, L-KM), medium (Medium-Koumine, M-KM), and high (High-Koumine, H-KM) concentrations of koumine, exhibiting a clear dose-dependent inhibitory effect. The positive control drug oxaliplatin (L-OHP) also significantly suppressed colony formation (**Figure 4B**).

To determine whether the growth-inhibitory effects of koumine were associated with apoptosis induction, flow cytometry analysis was conducted. As shown in **Figure 4C**, treatment with low, medium, and high concentrations of koumine for 48 h significantly increased the apoptosis rates of both HCT15 and HCT116 cells compared with the control group, with apoptotic effects intensifying in a concentration-dependent manner. Oxaliplatin treatment similarly induced apoptosis. Furthermore, Western blot analysis was used to examine the expression of apoptosis-related proteins (**Figure 4D**). Following koumine treatment, the expression of the anti-apoptotic protein Bcl-2 was significantly downregulated, whereas the pro-apoptotic protein Bax was markedly upregulated in both cell lines. These findings suggest that koumine promotes apoptosis in colorectal cancer cells by regulating Bcl-2 and Bax expression.

### Koumine inhibits cell migration and invasion of colorectal cancer

**Figure 5:**
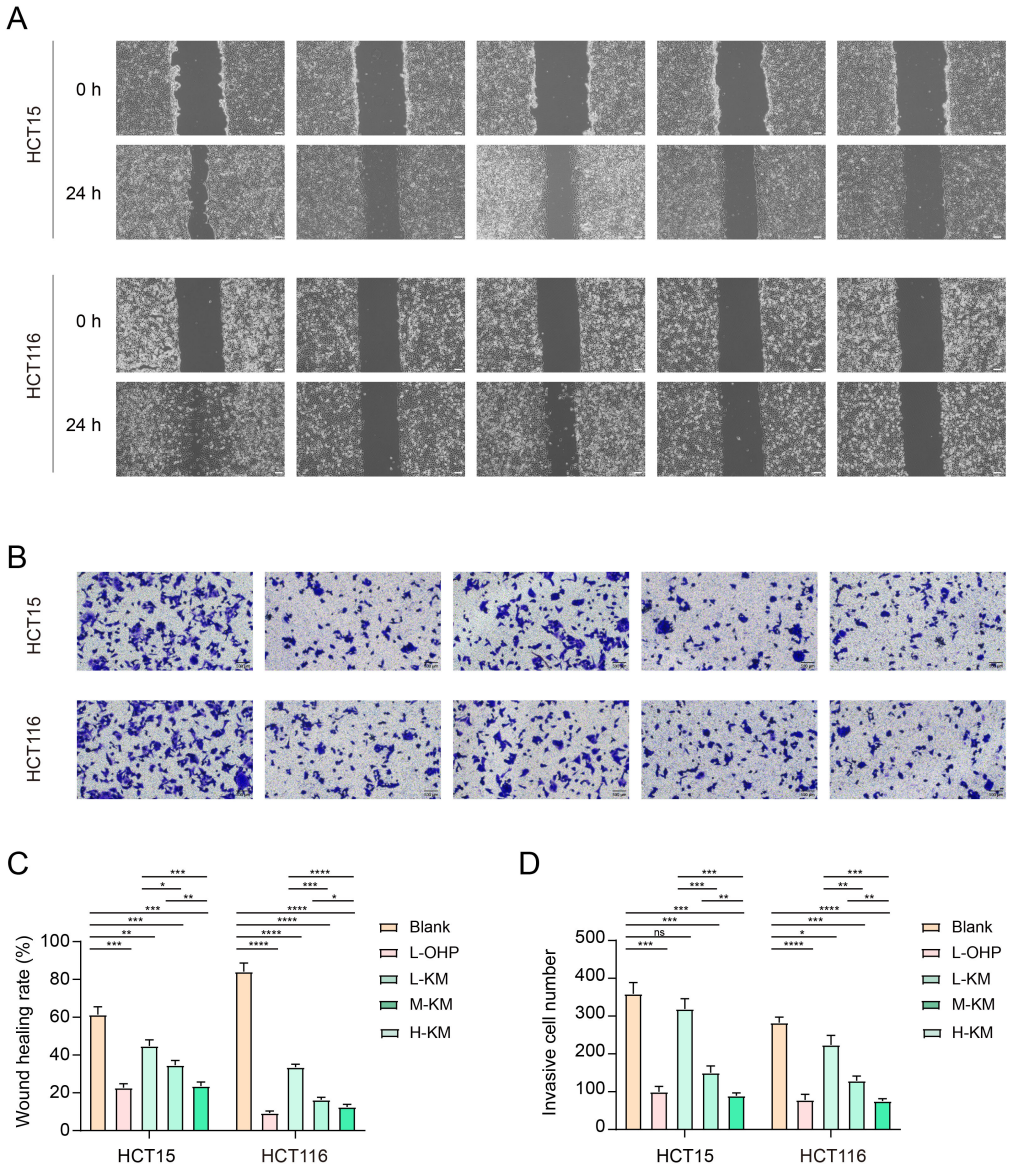
Koumine inhibits migration and invasion of colorectal cancer cells. **(A)** Scratch wound healing assay in HCT15 and HCT116 cells treated with koumine (100, 200, and 400 µg/mL) or the positive control L-OHP (5 µg/mL). **(B)** Transwell invasion assay of HCT15 and HCT116 cells treated with koumine (100, 200, and 400 µg/mL) or L-OHP (5 µg/mL). **(C)** Quantitative analysis of wound healing area in the scratch assay. **(D)** Quantitative analysis of invaded cells in the lower chamber of the Transwell assay. All experiments were independently repeated three times, and data are presented as mean ± SD. *P < 0.05, **P < 0.01, ***P < 0.001, ****P < 0.0001.

To evaluate the effects of koumine on the migratory and invasive abilities of colorectal cancer cells, scratch wound healing and Transwell invasion assays were performed. In the scratch wound healing assay (**Figure 5A**), after 24 h of treatment with koumine at low (100 μg/mL, L-KM), medium (200 μg/mL, M-KM), and high (400 μg/mL, H-KM), both HCT15 and HCT116 cells exhibited significantly reduced wound closure in a dose-dependent manner. Quantitative analysis showed that the M-KM group displayed the lowest wound healing rate, indicating that the medium concentration of koumine exerted the strongest inhibitory effect on cell migration (**Figure 5C**). In the Transwell invasion assay (**Figure 5B**), cells cultured in the upper chamber with serum-free medium and treated with koumine demonstrated a marked reduction in the number of cells migrating through the Matrigel-coated membrane into the lower chamber compared with the control group. The inhibitory effect on cell invasion increased with rising concentrations of koumine, with the strongest suppression observed in the H-KM group. The positive control, oxaliplatin (L-OHP), also significantly reduced cell invasion. Quantitative analysis (**Figures 5D**) further confirmed that koumine significantly decreased the number of invasive cells in a dose-dependent manner in both HCT15 and HCT116 cells. Collectively, these results indicate that koumine effectively inhibits colorectal cancer cell migration and invasion.

### By targeting HSP90, koumine disrupts its interaction with CDC37 and inhibits downstream signaling pathways

Molecular docking analysis predicted a potential interaction between koumine and HSP90. However, due to the large disparity in molecular weight between HSP90 and koumine, accurate determination of their binding affinity using surface plasmon resonance (SPR) was technically challenging because of ligand-coupling constraints. Therefore, the cellular thermal shift assay (CETSA) was employed to further examine this interaction. The results demonstrated that koumine treatment induced a pronounced shift in the thermal denaturation curve of HSP90, with a ΔTm_50_ ranging from 3.48°C to 12.01°C, indicating increased protein thermal stability and supporting a direct binding interaction between koumine and HSP90.

**Figure 6:**
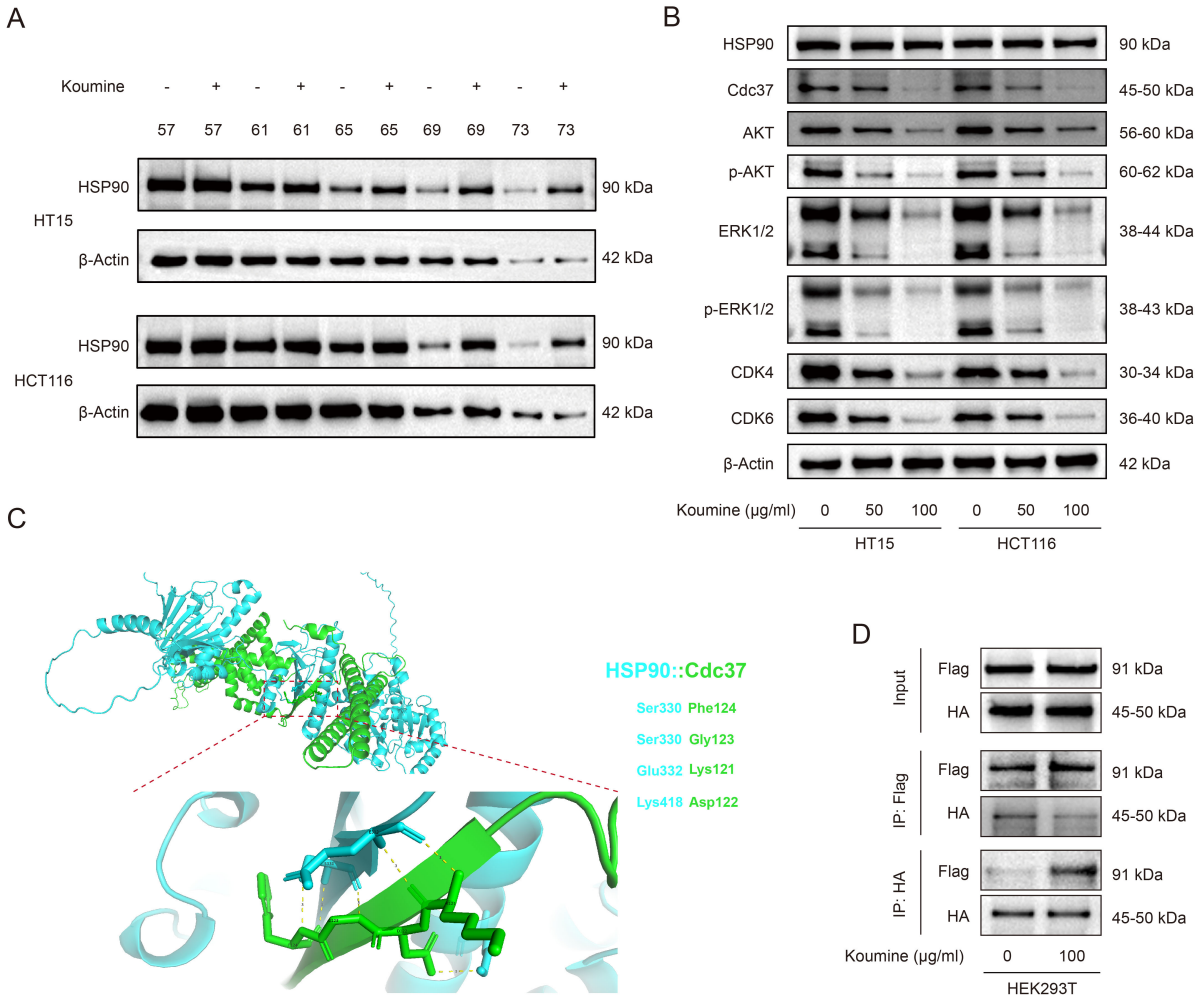
Western blot analysis reveals that koumine inhibits the HSP90/CDC37 complex and downstream AKT signaling. **(A)** Western blot analysis of HSP90 expression in HCT15 and HCT116 cells treated with koumine (0, 50, and 100 μg/mL). **(B)** Western blot analysis of AKT signaling pathway–related proteins in HCT15 and HCT116 cells treated with koumine (0, 50, and 100 μg/mL). **(C)** Molecular docking analysis of the HSP90–CDC37 interaction. The predicted binding mode highlights key residues involved in complex formation, including Ser330, Glu332, Lys121, and Lys418, supporting the hypothesis that koumine disrupts the HSP90–CDC37 interaction. **(D)** Co-immunoprecipitation (Co-IP) assay in HEK293T cells confirming the inhibitory effect of koumine on the HSP90–CDC37 interaction.

Previous studies have shown that disruption of the HSP90–CDC37 complex can suppress downstream PI3K/AKT signaling (21, 22). Western blot analysis revealed that koumine treatment did not significantly alter total HSP90 protein levels in HCT15 and HCT116 cells (**Figure 6A**), suggesting that koumine modulates HSP90 function rather than its expression. We next assessed the impact of koumine on AKT pathway activation. Western blot results showed that koumine suppressed both the expression and phosphorylation of AKT and ERK1/2, which was accompanied by reduced expression of downstream cell cycle regulators CDK4 and CDK6 (23) (**Figure 6B**; **Supplementary Figure S1**). These findings indicate that koumine inhibits AKT-related signaling pathways through functional interference with HSP90, thereby restraining colorectal cancer cell proliferation and mitotic activity.

To further investigate the mechanism by which koumine interferes with HSP90 function, molecular docking analysis to model the interaction between HSP90 and its co-chaperone CDC37. The predicted binding interface highlighted several key residues—including Ser330, Glu332, Lys121, and Lys418—that are critical for HSP90–CDC37 complex formation (**Figure 6C**), suggesting that koumine may disrupt this interaction. To experimentally validate this hypothesis, co-immunoprecipitation assays were conducted in HEK293T cells co-expressing Flag-tagged HSP90 and HA-tagged CDC37. Treatment with 100 μg/mL koumine markedly reduced the binding between HSP90 and CDC37 (**Figure 6D**). Together, these results demonstrate that koumine disrupts the HSP90–CDC37 interaction, leading to inhibition of downstream oncogenic signaling pathways.

### Effect of Koumine on tumor growth and apoptosis in colorectal cancer xenografts

**Figure 7:**
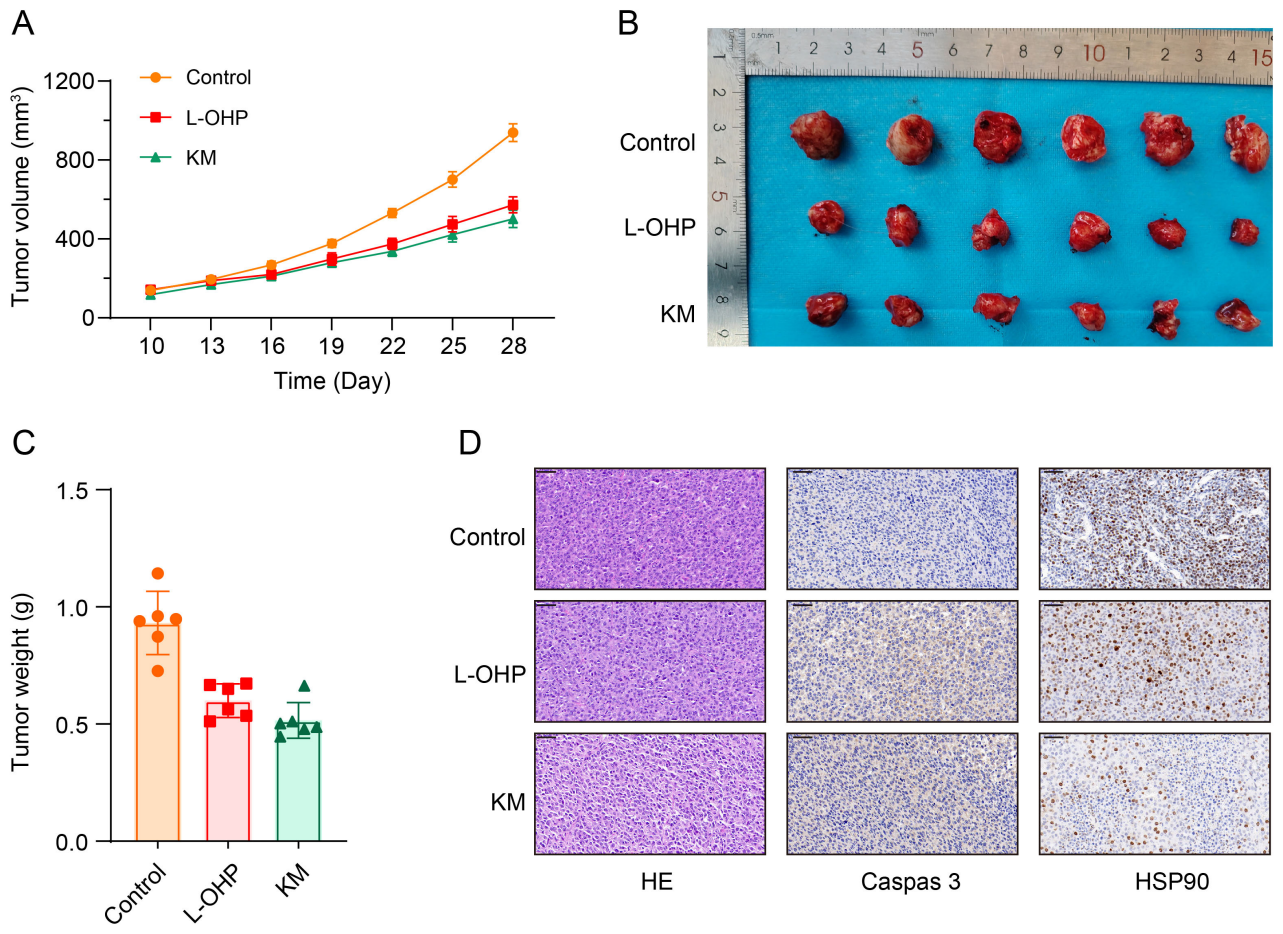
Effect of koumine on tumor growth and apoptosis in colorectal cancer xenografts. **(A)** Tumor volume measured over a 28-day period in BALB/c nude mice (n = 18) subcutaneously injected with 5 × 10^6^ HCT116 cells in 200 μL. Mice were treated with L-OHP (8 mg/kg, every 2 days), koumine (KM; 10 mg/kg, daily), or vehicle (control). **(B)** Representative images of tumors excised at the end of the experiment. **(C)** Tumor weights measured after euthanasia. **(D)** Histopathological analysis of tumor tissues using hematoxylin and eosin (H&E) staining and immunohistochemical staining for caspase-3 and HSP90. P < 0.05, *P < 0.01, **P < 0.001.

Tumor over a 28-day treatment period in control, oxaliplatin (L-OHP), and koumine (KM) treatment groups. As shown in **Figure 7A**, tumor growth in the control group was significantly faster than in both the L-OHP and KM groups. Koumine treatment resulted in a marked inhibition of tumor growth, with significantly reduced tumor volumes compared with the control group. Representative images of excised tumors further confirmed the reduced tumor size in the KM- and L-OHP-treated groups relative to controls (**Figure 7B**). Tumor weight analysis demonstrated that tumors from the KM-treated group weighed significantly less than those from the control group, with a comparable reduction observed in the L-OHP group (**Figure 7C**). Histopathological examination using hematoxylin and eosin staining, along with immunohistochemical analysis of caspase-3 and HSP90 expression, was performed to assess tumor apoptosis and molecular changes (**Figure 7D**). Tumors from the KM-treated group exhibited stronger caspase-3 staining, indicating enhanced apoptosis, whereas weak caspase-3 expression was observed in control tumors. In addition, HSP90 expression was reduced in the KM-treated group, consistent with the proposed mechanism of koumine action. These results demonstrate that koumine suppresses colorectal cancer xenograft growth by promoting apoptosis and targeting HSP90-mediated signaling pathways.

## Discussion

The traditional use of the Chinese herb *Gelsemium* for tumor treatment has been documented in classical Chinese medical texts. Modern pharmacological studies have demonstrated that extracts from *Gelsemium* can inhibit tumor cell proliferation and induce apoptosis (24). With advances in analytical technologies, the primary bioactive components of *Gelsemium* extracts have been successfully isolated and identified (7). Among these, koumine is a monoterpene alkaloid derived from the total alkaloids of *Gelsemium*, accounting for approximately 30% of the total alkaloid content (25). Koumine represents the principal component of *Gelsemium* alkaloids and exhibits comparable biological activity with relatively lower toxicity, highlighting its potential clinical value (26). Preclinical studies have demonstrated the therapeutic effects of koumine in neuropathic pain (27), arthritis (28), and autoimmune liver injury (29). In the context of cancer, koumine has shown marked cytotoxic effects against leukemia, liver cancer, breast cancer, lung cancer, and colon cancer cells (12, 13). However, the molecular mechanisms underlying its anticancer activity remain insufficiently characterized.

In the initial phase of this study, network pharmacology was applied to identify intersecting targets between koumine and colorectal cancer–associated proteins, yielding 65 overlapping candidates. Protein–protein interaction network analysis revealed that STAT3 and HSP90AB1 exhibited high connectivity within the network, suggesting that they may serve as key downstream targets of koumine. Subsequent pathway enrichment analysis indicated that koumine may regulate oxidative stress–related pathways, angiogenesis-related processes, tyrosine kinase activity, and transcriptional regulation, reflecting its broad modulatory effects on cellular signaling. Strategies such as enhancing oxidative stress (30), inhibiting angiogenesis (31), suppressing tyrosine kinase activity (32), and limiting aberrant transcriptional activity (33) are well-recognized approaches in colorectal cancer therapy, further supporting the potential anticancer value of koumine.

To clarify the downstream mechanisms of koumine, molecular docking analyses were performed to predict interactions between koumine and the identified intersecting targets. Among these, ABL1, CXCR4, JAK2, JAK1, STAT3, and HSP90AB1 demonstrated the strongest predicted binding affinities, indicating potential direct interactions. These in silico findings were subsequently supported by comprehensive *in vitro* and *in vivo* experiments. In colorectal cancer cell models, CCK-8 and colony formation assays confirmed the dose-dependent inhibitory effects of koumine on cell proliferation, while flow cytometry demonstrated its ability to induce apoptosis. Moreover, wound healing and Transwell invasion assays showed that koumine significantly suppressed the migratory and invasive capacities of colorectal cancer cells. Consistently, the xenograft mouse model revealed that koumine markedly inhibited tumor growth, with antitumor efficacy comparable to that of the clinically used chemotherapeutic agent oxaliplatin (L-OHP). Collectively, these findings provide robust evidence supporting koumine as a promising therapeutic agent against colorectal cancer.

**Figure 8:**
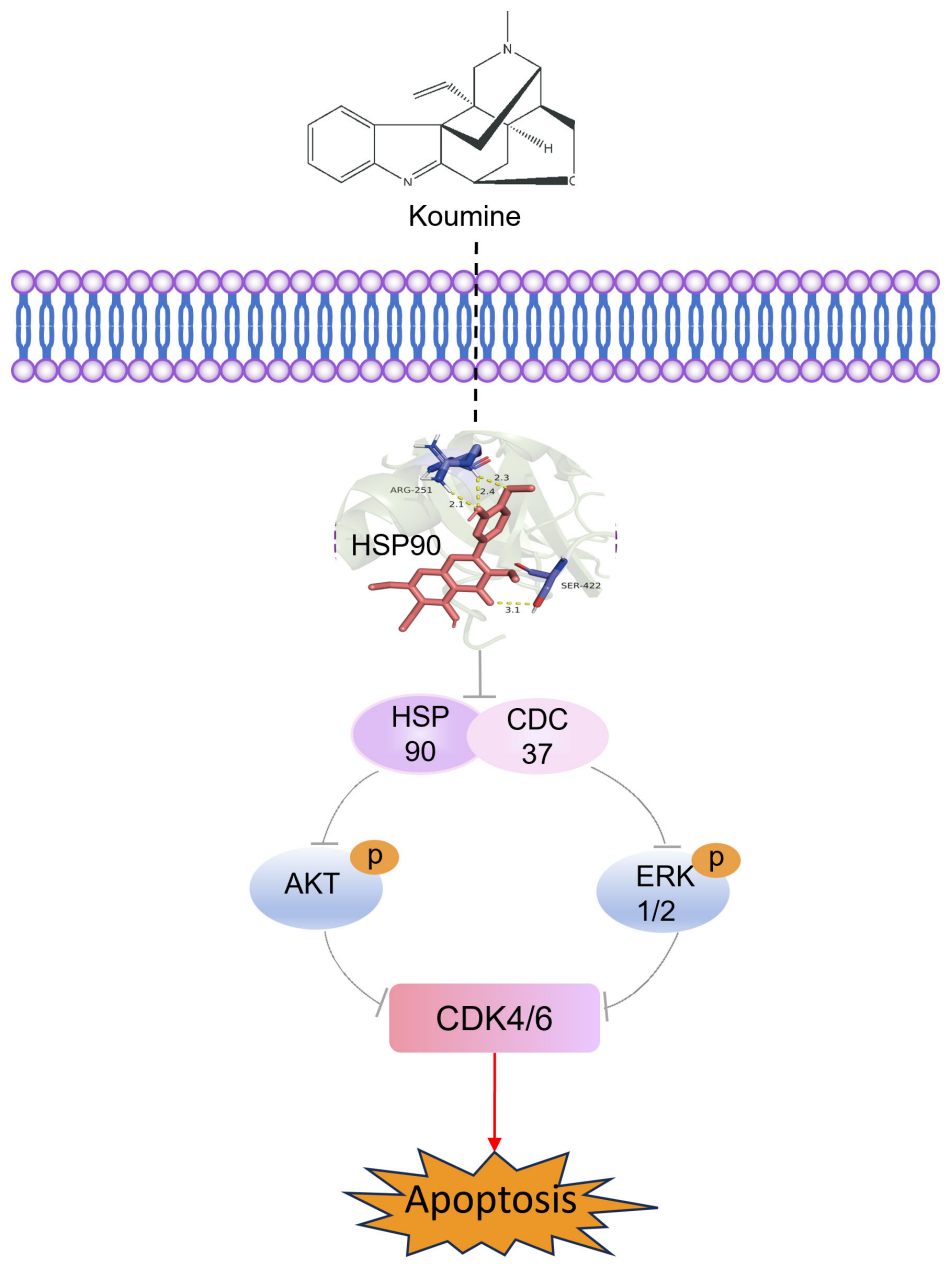
Mechanism of koumine-induced apoptosis in colorectal cancer cells. Schematic illustration of the proposed mechanism by which koumine induces apoptosis in colorectal cancer cells. Koumine targets HSP90 and disrupts its interaction with CDC37, leading to inactivation of the AKT and ERK1/2 signaling pathways and subsequent downregulation of CDK4 and CDK6. These molecular events collectively result in the induction of apoptosis, highlighting the therapeutic potential of koumine in colorectal cancer.

Furthermore, we conducted preliminary investigations into the mechanism of action of koumine on colorectal cancer. Based on the stabilization of the target protein when it binds to the drug molecule, CETSA is applied for evaluating the binding efficiency of a drug to its target protein within cells (34, 35). As the temperature increases, proteins generally denature and degrade; while the amount of intact protein at a given temperature increases when bound to a drug, resulting in a rightward shift in the protein’s thermal melting curve. Through CETSA experiments, we discovered that the addition of koumine enhances the thermal stability of HSP90. This observation indicates a direct interaction between koumine and HSP90. HSP90 functions as a molecular chaperone that facilitates the folding and maturation of numerous client proteins through ATP hydrolysis within a multichaperone complex that includes the kinase-specific co-chaperone CDC37 (22, 36). CDC37 plays a pivotal role in recruiting kinase clients to HSP90 by interacting with both the client proteins and HSP90 itself (37). Disruption of the HSP90–CDC37 interaction leads to destabilization and accelerated degradation of downstream client proteins, ultimately impairing tumor cell proliferation (38). Targeting this protein–protein interaction has emerged as an innovative anticancer strategy, and several HSP90–CDC37 inhibitors have demonstrated significant tumor-suppressive effects in colorectal cancer (39–41) models. Consistent with this mechanism, Western blot analyses revealed that koumine modulated CDC37 expression and suppressed both the expression and phosphorylation of AKT and ERK1/2. This signaling inhibition ultimately led to reduced expression of the cell cycle regulators CDK4 and CDK6 (42–45), which are critical for cell cycle progression and mitotic activity (23). Together, these findings elucidate a coherent molecular pathway through which koumine exerts its anti‑colorectal cancer effects, as summarized in **Figure 8**.

## Conclusion

Koumine exerts antitumor effects against colorectal cancer by targeting HSP90 and disrupting its interaction with CDC37. This disruption leads to inhibition of AKT/ERK signaling and downregulation of CDK4 and CDK6, resulting in suppressed tumor cell proliferation, impaired migration and invasion, and enhanced apoptosis. Supported by both *in vitro* and *in vivo* evidence, this study identifies koumine as a promising therapeutic candidate for colorectal cancer and provides a mechanistic foundation for its potential development in anticancer therapy.

## Data Availability

The original contributions presented in the study are included in the article/**Supplementary Material**. Further inquiries can be directed to the corresponding authors.

## Supplementary material

Supplementary Figure 1 Statistical analysis of protein expression from Western blot experiments. Bar graphs show protein expression levels in HCT15 (left) and HCT116 (right) cells treated with different concentrations of koumine (KM): KM-0 (control), KM-50 (50 μg/mL), and KM-100 (100 μg/mL). Expression levels of HSP90, CDC37, AKT, p-AKT, ERK1/2, p-ERK1/2, CDK4, and CDK6 were analyzed by Western blot and normalized to β-actin. Data are presented as mean ± SD from three independent experiments. P < 0.05, *P < 0.01, **P < 0.001.
