# Supplementary figures and images for: Koumine exerts its anti-colorectal cancer effects by disrupting the interaction between HSP90 and CDC37, thereby downregulating downstream signaling pathways
Source: Front Oncol. 2026 Jan 19;15:1687690. doi: 10.3389/fonc.2025.1687690 (PMC12861894; doi:10.3389/fonc.2025.1687690)

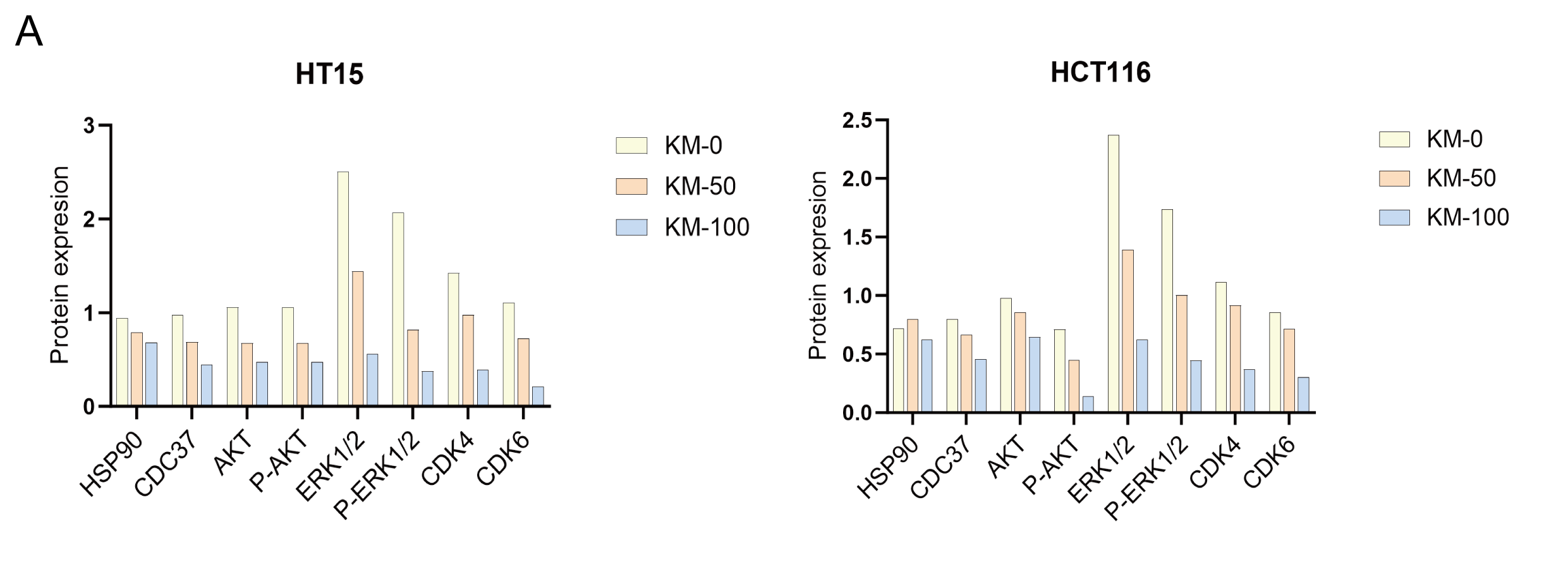

Supplement: Supplementary Figure 1 — Statistical analysis of protein expression from Western blot experiments. Bar graphs show protein expression levels in HCT15 (left) and HCT116 (right) cells treated with different concentrations of koumine (KM): KM-0 (control), KM-50 (50 μg/mL), and KM-100 (100 μg/mL). Expression levels of HSP90, CDC37, AKT, p-AKT, ERK1/2, p-ERK1/2, CDK4, and CDK6 were analyzed by Western blot and normalized to β-actin. Data are presented as mean ± SD from three independent experiments. P < 0.05, *P < 0.01, **P < 0.001. [file Image1.tif]
