# Supplementary material for: Koumine exerts its anti-colorectal cancer effects by disrupting the interaction between HSP90 and CDC37, thereby downregulating downstream signaling pathways
Source: Front Oncol. 2026 Jan 19;15:1687690. doi: 10.3389/fonc.2025.1687690 (PMC12861894; doi:10.3389/fonc.2025.1687690)
Supplement: Supplementary file 2 [file Table1.docx]

**Table S1 Parameters of the docking box**

| **Target Name** | **Center_x** | **Center_y** | **Center_z** | **size** **_x** | **size** **_y** | **size** **_z** |
| --- | --- | --- | --- | --- | --- | --- |
| ABL1 | -26.228 | 29.886 | -15.157 | 126 | 126 | 126 |
| CXCR4 | 36.255 | 4.164 | 20.843 | 126 | 126 | 126 |
| JAK2 | 97.242 | 73.728 | 12.715 | 126 | 126 | 126 |
| JAK1 | 8.242 | 27.746 | 9.715 | 126 | 126 | 126 |
| STAT3 | -1.655 | 14.616 | 23.599 | 126 | 126 | 126 |
| HSP90AB1 | 1.843 | 16.011 | 20.904 | 126 | 126 | 126 |
