# Supplementary material for: Koumine exerts its anti-colorectal cancer effects by disrupting the interaction between HSP90 and CDC37, thereby downregulating downstream signaling pathways
Source: Front Oncol. 2026 Jan 19;15:1687690. doi: 10.3389/fonc.2025.1687690 (PMC12861894; doi:10.3389/fonc.2025.1687690)
Supplement: Supplementary file 3 [file Table2.docx]

**Table S2 Antibodies information**

| **Protein Symbol** | **Dilution Ratio** | **Cat No.** | **MFRS.** | **Country** |
| --- | --- | --- | --- | --- |
| HSP90 | 1:5000 for WB | 13171-1-AP | Proteintech | China |
| CDC37 | 1:5000 for WB | ab109419 | Abcam | USA |
| AKT | 1:5000 for WB | 60203-2-Ig | Proteintech | China |
| p-AKT | 1:10000 for WB | 80455-1-RR | Proteintech | China |
| ERK1/2 | 1:2000 for WB | ab196883 | Abcam | USA |
| p-ERK1/2 | 1:200 for WB | ab278538 | Abcam | USA |
| CDK4 | 1:10000 for WB | 66950-1-Ig | Proteintech | China |
| CDK6 | 1:1000 for WB | AF2536 | Beyotime | China |
| β-actin | 1:1000 for WB | AF0003 | Beyotime | China |
| Bax | 1:1000 for WB | ab182734 | Abcam | USA |
| Bcl2 | 1:2000 for WB | ab238041 | Abcam | USA |
| Caspase3 | 1:200 for IHC | 19677-1-AP | Proteintech | China |
| HSP90 | 1:4000 for IHC | 13171-1-AP | Proteintech | China |
